# Comparison of PD-L1 expression in head and neck squamous cell carcinoma among preoperative biopsy, surgical resection and metastatic lymph node

**DOI:** 10.3389/fonc.2025.1666078

**Published:** 2025-10-15

**Authors:** Heeseung Sohn, Meejeong Kim, Sang-Yeon Kim, Dong-Il Sun, Youn Soo Lee

**Affiliations:** ^1^ Department of Hospital Pathology, College of Medicine, The Catholic University of Korea, Seoul, Republic of Korea; ^2^ Department of Otorhinolaryngology-Head and Neck Surgery, College of Medicine, The Catholic University of Korea, Seoul, Republic of Korea

**Keywords:** head and neck squamous cell carcinoma, PD-L1, combined positive score, tumor proportion score, heterogeneity

## Abstract

**Background:**

The interpretation of PD-L1 expression in advanced head and neck squamous cell carcinoma (HNSCC) has recently emerged as a component of companion diagnostics, essential in identifying candidates for immune checkpoint inhibitors (ICIs). However, evaluating PD-L1 expression using the combined positive score (CPS) and tumor proportion score (TPS) has posed challenges, particularly in selecting appropriate specimens and determining hotspots for measurement.

**Methods:**

This study included 68 HNSCC cases from the oropharynx and tongue, collecting complete sets of preoperative biopsy, surgical resection, and metastatic lymph node samples. PD-L1 22C3 staining was performed on each 204 samples to assess PD-L1 expression. The CPS and TPS were measured in all samples using QuPath, an open-source bioimage analysis tool. Statistical comparisons of CPS and TPS among the three specimen types were conducted using Kruskal-Wallis test.

**Results:**

Significant discrepancies were observed in both CPS and TPS between biopsy and surgical resection (p <0.01, respectively), as well as in both CPS and TPS between resection and metastatic lymph node (p <0.01, respectively). However, no significant statistical differences were observed between the biopsy and metastatic lymph node.

**Conclusions:**

Our analysis highlights the heterogeneity of PD-L1 expression across different specimen types. Significant variability exists between the preoperative biopsy and surgical resection, and between surgical resection and metastatic lymph node in patients with HNSCC. These findings suggest that PD-L1 expression varies in response to tumor microenvironment across various tumor areas and time frames. Therefore, individualized PD-L1 assessment for each specimen type is crucial for accurately determining eligibility for ICIs.

## 1 Introduction

Head and neck cancer is the seventh most common type of malignancy, with approximately 890,000 new cases diagnosed worldwide annually (1). Over 90% of these cases are classified as squamous cell carcinoma. Head and neck squamous cell carcinoma (HNSCC) significantly impairs a patient’s quality of life, with a high risk of metastasis or recurrence, often leading to poor clinical outcomes despite multimodal treatment strategies.

Programmed death-ligand 1 (PD-L1) is an immune checkpoint protein expressed on tumor cells. Its interaction with PD-L1 receptors on T cells results in immune tolerance, allowing tumor cells to evade immune surveillance. Several studies that have reported an association between PD-L1 expression and clinical factors in HNSCC, with some suggesting that the presence of human papilloma virus (HPV) is associated with higher PD-L1 expression likely due to immune activation (2).

The introduction of immune checkpoint inhibitors (ICIs) has revolutionized cancer treatment by restoring T cell-mediated immune responses against tumor cells. ICIs, such as pembrolizumab, has been approved as both a first-line and second-line treatment options for advanced HNSCC. Accurate pathological assessment of PD-L1 expression, using the combined positive score (CPS) and tumor proportion score (TPS), remains critical for guiding patient selection.

Based on the KEYNOTE-689 trial, current guidelines recommend pembrolizumab for patients with resectable, locally advanced HNSCC (stage III–IVA) when CPS is ≥1 in the first-line setting and TPS is ≥50% in the second-line setting (3). In addition, the CHECKMATE-141 trial demonstrated that nivolumab is effective in recurrent HNSCC irrespective of PD-L1 expression or p16 status (4).

Many studies have examined PD-L1 expression levels in cohorts of patients with non-small cell lung cancer (NSCLC). Some have demonstrated intra-tumoral heterogeneity of PD-L1 expression (4), as well as inter-site heterogeneity within individual tumors (5, 6), raising the question of whether PD-L1 expression changes during tumor metastasis.

In the HNSCC, some contradictory reports suggest that a single specimen may adequately represent the PD-L1 expression of the entire tumor, regardless of the degree of intra-tumoral heterogeneity (7), while others indicate that recurrent or metastatic tumors may show distinct PD-L1 expression patterns compared to primary tumors (8).

Despite the clinical significance of PD-L1 expression, its consistency across different specimen types—including preoperative biopsy, surgical resection, and metastatic lymph nodes—remains controversial. However, there are no standardized guidelines for PD-L1 interpretation across different specimen types. There is no consensus on which tissue should best be used for PD-L1 immunohistochemical testing to avoid misclassification of PD-L1 status (9).

This study aims to investigate the heterogeneity of PD-L1 expression across different specimen types in HNSCC and to analyze the comparative relationships between CPS and TPS measurements.

## 2 Materials and methods

### 2.1 Patient and material

Between 2014 and 2023, histologically confirmed cases of HNSCC were retrieved from the pathology archives of the Department of Hospital Pathology, Seoul St. Mary’s Hospital. A total of 68 cases were retrospectively selected, comprising tumors of the oropharynx (palatine tonsil and base of tongue) and oral cavity (tongue proper and floor of mouth). These anatomical sites were selected for their accessibility and rising incidence, while tumors of the hypopharynx, maxilla, larynx were excluded due to limited case numbers and suboptimal PD-L1 staining following decalcification.

Within the oropharyngeal group (39 cases), 14 were stage I, 19 were stage II, five were stage III, and one was stage IV at diagnosis. In the oral cavity group (29 cases), 20 were stage III and nine were stage IV. For each case, complete sets of preoperative biopsy, surgical resection, and lymph node metastasis samples were collected. In most cases (66/68), lymph node metastases were resected during the same surgical procedure as the primary tumor. Hematoxylin and eosin (H&E)-stained slides were reviewed to confirm the presence of carcinoma.

### 2.2 Immunohistochemistry procedures

Initial HPV status screening was performed using p16 IHC. Confirmation was then conducted with the PANA RealTyper HPV kit (Panagene, Daejeon, Korea), a multiplex real-time PCR assay performed on DNA extracted from the tumor tissue. In parallel, PD-L1 IHC was performed using the PD-L1 IHC 22C3 pharmDx assay (Agilent/Dako, CA, USA).

Both p16 and PD-L1 22C3 IHC were conducted on 4-µm-thick sections prepared from formalin-fixed, paraffin-embedded (FFPE) blocks. The entire process for the 204 tissue sections (representing complete sets of specimens from 68 patients) were automated on the Ventana BenchMark ULTRA automated staining platform (Ventana Medical Systems, AZ, USA).

### 2.3 Whole slide image preparation

Whole-slide images of the PD-L1-stained slides were digitalized using the Philips Intellisite Pathology Solution platform. The images were then exported as high-resolution TIFF files for further bioimage analysis.

### 2.4 Bioimage analysis

**Figure 1:**
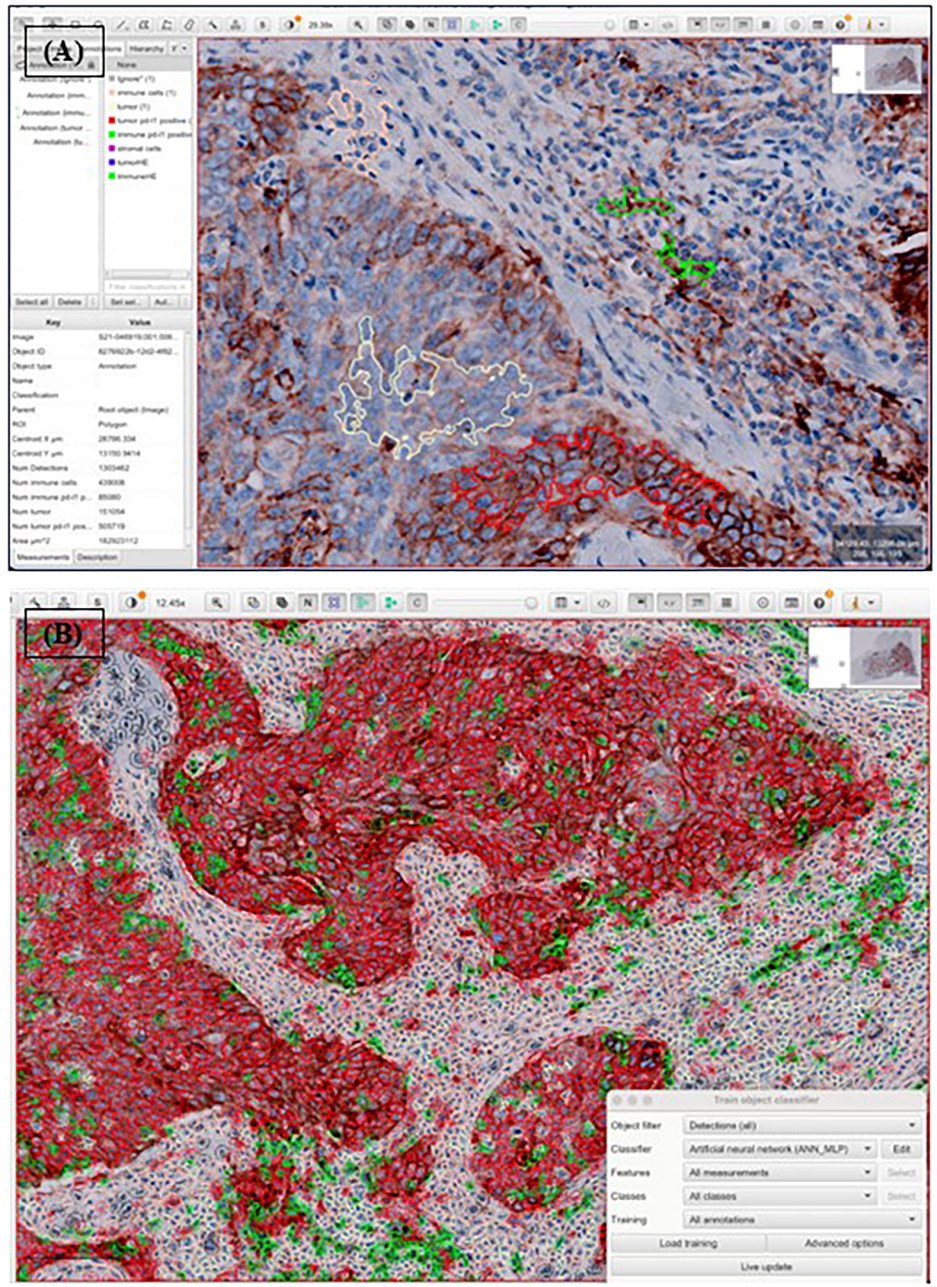
Procedure of automated analysis of PD-L1 22C3 expression using QuPath. Each PD-L1 22C3 pharmDx-stained slide was digitally scanned and exported into Qupath, followed by whole cell detection. Annotation was conducted to classify four different cell populations: tumor cells (light yellow), immune cells (pink), PD-L1 stained tumor cells (red), PD-L1 stained immune cells (green), non-relevant cells (black, not shown) **(A)**. The annotated classification was then applied across the entire slide using an object classifier **(B)**. CPS and TPS were subsequently calculated based on numerical outputs derived from the analysis.

QuPath, an open-source bioimage analysis platform, was used for the manual annotation of four distinct cell populations: tumor cells, immune cells, PD-L1-expressing tumor cells, and PD-L1-expressing immune cells (10) (**Figure 1**).

### 2.5 PD-L1 scoring and classification

**Figure 2:**
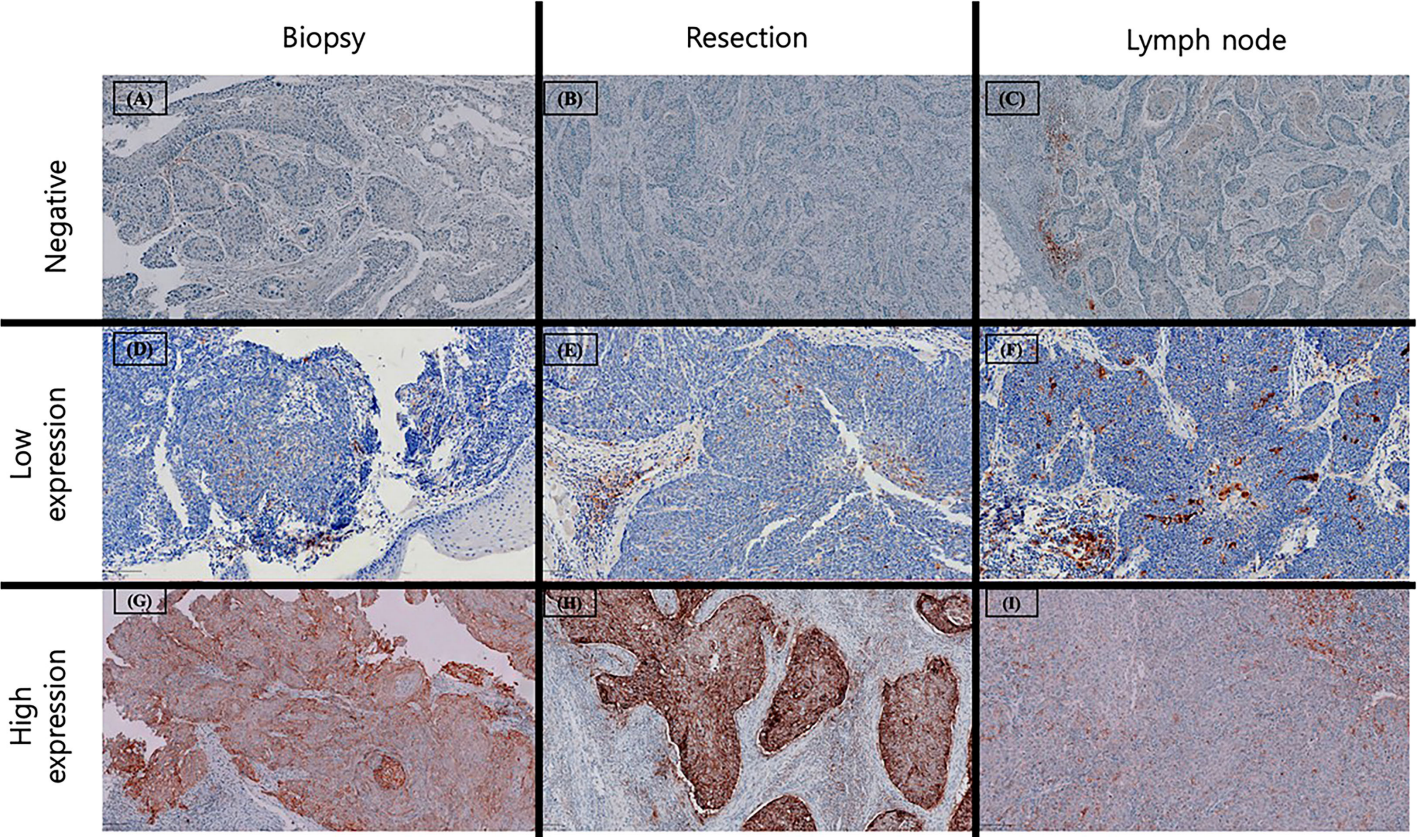
Examples of PD-L1 expression across different specimens. Negative PD-L1 expression (CPS < 1 or TPS < 1%) in preoperative biopsy **(A)**, surgical resection **(B)** and metastatic lymph node **(C)**. Low PD-L1 expression (CPS 1–20 or TPS 1- 50%) in preoperative biopsy **(D)**, surgical resection **(E)** and metastatic lymph node **(F)**, characterized by scarce staining in tumor cells with occasional PD-L1 stained mononuclear immune cells. High PD-L1 expression (CPS ≥ 20 or TPS ≥ 50%) in preoperative biopsy **(G)**, surgical resection **(H)** and metastatic lymph node **(I)** with strong staining in tumor cells and accompanying immune cells adjacent to tumor nests.

PD-L1 expression was evaluated using two standardized scoring systems for each specimen. The combined proportion score (CPS) was calculated as the number of PD-L1–positive cells, including tumor cells, lymphocytes, and macrophages, divided by the total number of viable tumor cells and multiplied by 100. The tumor proportion score (TPS) was calculated as the percentage of PD-L1–positive tumor cells among at least 100 viable tumor cells. Necrotic areas and non-neoplastic cellular components were excluded from the analysis. Based on these scores, specimens were classified into three expression categories: negative (CPS <1; TPS <1%), low expression (CPS 1–19; TPS 1–49%), and high expression (CPS ≥20; TPS ≥50%) (**Figure 2**).

### 2.6 Statistical analysis

Statistical analyses were performed to investigate the relationships between different variables. The Mann-Whitney U test was used to analyze the association between HPV status (determined by p16 IHC and PCR) and PD-L1 expression, as well as the association between PD-L1 expression and the stage of oropharyngeal cancer. The Kruskal-Wallis test was applied to compare CPS and TPS values across different specimen types (biopsy, resection, and metastasis). A two-tailed significance level of p<0.05 was used to determine statistical significance.

## 3 Results

### 3.1 Clinicopathological features

Among the 68 HNSCC cases, primary tumors originated from the oropharynx (57.3%, 39/68) and the oral cavity (42.7%, 29/68). The mean age at diagnosis was 59.1 years, with a male predominance (male 71%, female 29%). In oropharyngeal HNSCCs, HPV positivity was confirmed in 89.7% of cases (35/39) and was significantly associated with p16 IHC (p <0.05). CPS was significantly higher in HPV-positive oropharyngeal cancers in both biopsy and resection specimens (p < 0.05). Notably, CPS scores differed significantly between early-stage (I-II) and advanced-stage (III-IV) oropharyngeal cancers in resection specimens (p <0.05). No oral cavity HNSCCs were HPV-associated (0/29). Other clinicopathological aspects and PD-L1 scores are summarized in **Table 1**.

**Table 1:** Clinicopathologic characteristics.

Variable	Oropharynx (n=39)	Oral cavity (n=29)	All cases (n = 68)
Mean age (years)	60.0 (range: 45 – 83)	54. 0(range: 33 – 80)	59.1 (range: 33 – 83)
Sex (n, %)			
Male	30 (76.9%)	18 (62.1%)	48 (70.6%)
Female	9 (23.1%)	11 (37.9%)	20 (29.4%)
Human papilloma virus status (n, %)			
Positive	35 (89.7%)	0 (0%)	35 (51.5%)
Negative	4 (10.3%)	29 (100%)	33 (48.5%)
p16 status (n, %)			
Positive	37 (94.9%)	6 (20.7%)	43 (63.2%)
Negative	2 (5.1%)	23 (79.3%)	25 (36.8%)
Stage of the disease (n, %)			
I	14 (35.9%)	0 (0%)	
II	19 (48.7%)	0 (0%)	
III	5 (12.8%)	20 (69.0%)	
IV	1 (2.6%)	9 (31.0%)	
CPS (Combined positive score) (n, %)			
Preoperative biopsy			
<1	2 (5.1%)	1 (3.4%)	3 (4.4%)
1-19	11 (28.2%)	10 (34.5%)	21 (30.9%)
≥ 20	26 (66.7%)	18 (62.1%)	44 (64.7%)
Surgical resection			
<1	0 (0.0%)	0 (0.0%)	0 (0%)
1-19	5 (12.8%)	4 (13.8%)	9 (13.2%)
≥ 20	34 (87.2%)	25 (86.2%)	59 (86.8%)
Metastatic lymph node			
<1	1 (2.6%)	1 (3.4%)	2 (2.9%)
1-19	11 (28.2%)	9 (31.0%)	20 (29.4%)
≥ 20	27 (69.2%)	19 (65.5%)	46 (67.7%)
TPS (Tumor proportion score) (n, %)			
Preoperative biopsy			
<1	5 (12.8%)	4 (13.8%)	9 (13.2%)
1-49	26 (66.7%)	18 (62.1%)	44 (64.7%)
≥ 50	8 (20.5%)	7 (24.1%)	15 (22.1%)
Surgical resection			
<1	0 (0.0%)	1 (3.4%)	1 (1.5%)
1-49	26 (66.7%)	16 (55.2%)	42 (61.7%)
≥ 50	13 (33.3%)	12 (41.4%)	25 (36.8%)
Metastatic lymph node			
<1	6 (15.4%)	5 (17.2%)	11 (16.2%)
1-49	21 (53.8%)	19 (65.5%)	40 (58.8%)
≥ 50	12 (30.8%)	5 (17.2%)	17 (25.0%)

### 3.2 PD-L1 expression in general

The average CPS and TPS was 42.8 (0.3-100) and 27.8% (0-92.1) in preoperative biopsies, 62.2 (4.1 -100) and 40.7% (0.04-100) in surgical resections, and 47.4 (0.1-100) and 27.9% (0-9-2.7) in metastatic lymph nodes.

In preoperative biopsies, CPS was negative in 3/68 (4.4%), low in 21/68 (30.9%), and high in 44/68 (64.7%). TPS was negative in 9/68 (13.2%), low in 44/68 (64.7%), and high in 15/68 (22.1%). In surgical resections, CPS was negative in 0/68 (0%), low in 9/68 (13.2%), and high in 59/68 (86.8%). TPS was negative in 1/68 (1.5%), low in 42/68 (61.7%), and high in 25/68 (36.8%). In metastatic lymph nodes, CPS was negative in 2/68 (2.9%), low in 20/68 (29.4%), and high in 46/68 (67.7%). TPS was negative in 11/68 (16.2%), low in 40/68 (58.8%), and high in 17/68 (25.0%).

Among PD-L1–positive cases (low and high expression), CPS consistently showed higher expression in oropharyngeal cancers across all specimen types, whereas TPS displayed variable patterns between oropharyngeal and oral cavity tumors.

**Figure 3:**
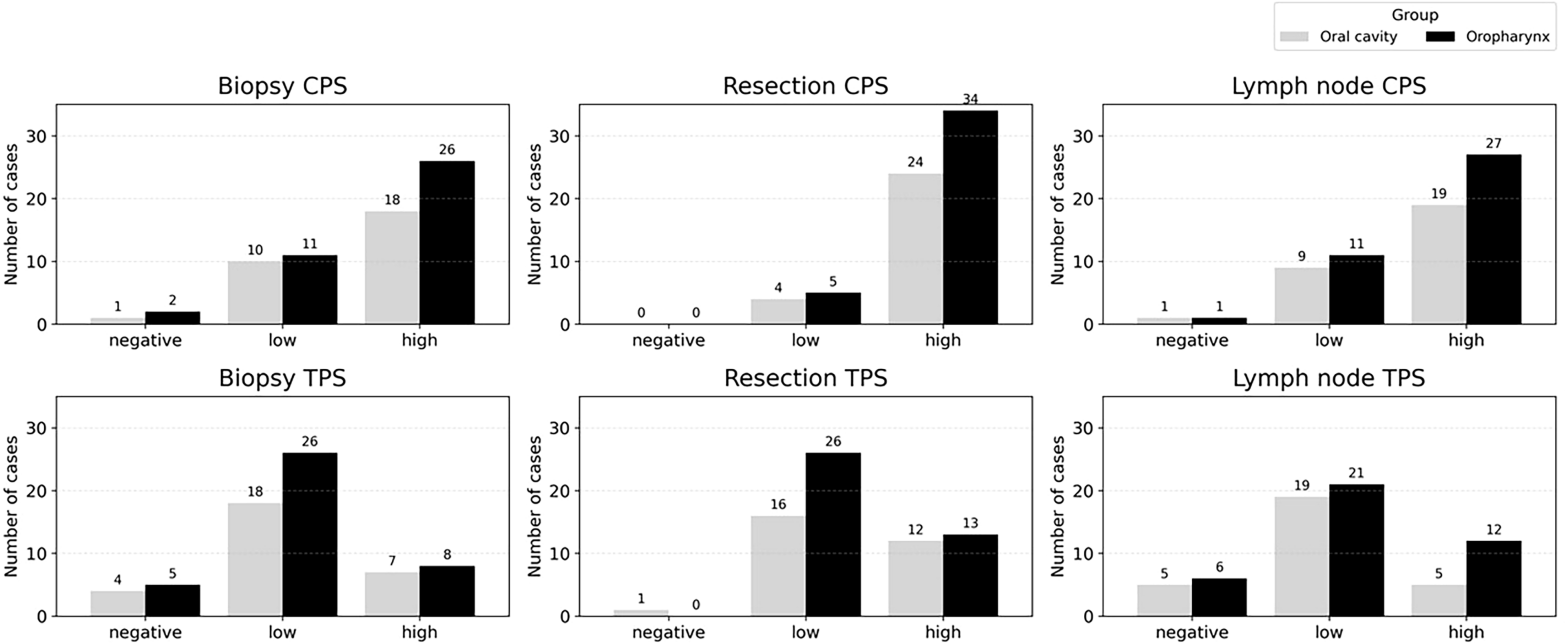
PD-L1 expression by CPS (top) and TPS (bottom) in oropharyngeal and oral cavity carcinomas across biopsy, resection, and lymph node specimens. CPS and TPS were categorized as negative (CPS < 1, TPS < 1%), low (CPS 1–19, TPS 1–49%), and high (CPS ≥ 20, TPS ≥ 50%).

In preoperative biopsies, CPS positivity was observed in 26/37 (70.3%) oropharyngeal cancers and 18/28 (64.3%) oral cavity cancers, while TPS positivity was found in 8/34 (23.5%) and 7/25 (28.0%), respectively. In surgical resections, CPS positivity was seen in 34/39 (87.2%) oropharyngeal cancers and 25/29 (86.2%) oral cavity cancers, with TPS positivity in 13/39 (33.3%) and 12/28 (42.9%), respectively. In metastatic lymph nodes, CPS positivity was present in 27/38 (71.1%) oropharyngeal cancers and 19/28 (67.9%) oral cavity cancers, while TPS positivity was seen in 12/33 (36.4%) and 5/24 (20.8%), respectively. Comparison bar graphs for oropharyngeal and oral cavity tumors across specimen types are shown in **Figure 3**.

### 3.3 HPV status and PD-L1 expression

**Figure 4:**
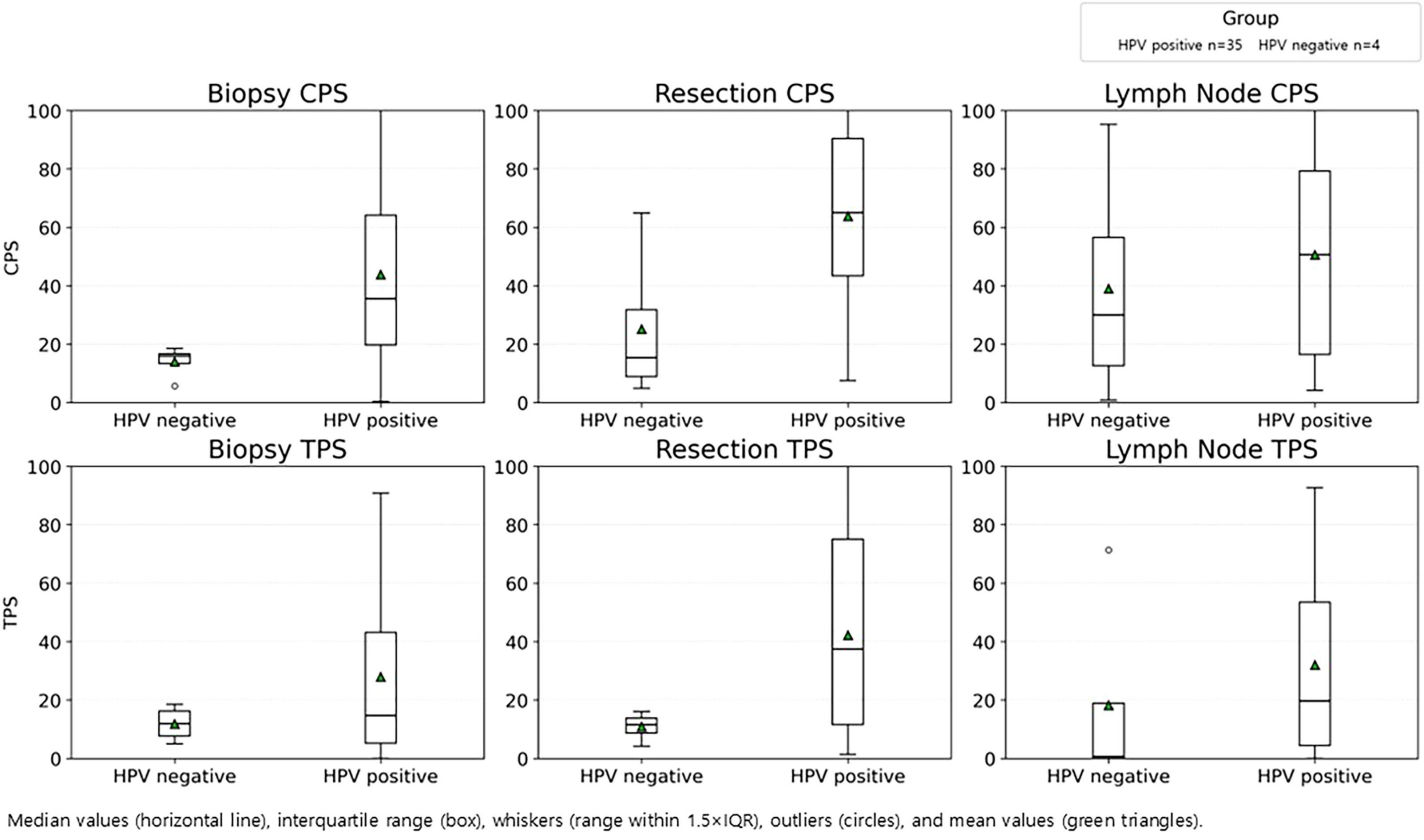
PD-L1 expression by HPV status in oropharyngeal cancer. CPS (top) and TPS (bottom) in biopsy, resection, and lymph node specimen, stratified by HPV-positive (n=35) and HPV-negative (n=4) groups.

In oropharyngeal cancers (n=39), HPV-positive tumors were associated with significantly higher PD-L1 expressions than HPV-negative cases for CPS for biopsy and resection specimens, and for TPS in resection specimens (p <0.05) (**Figure 4**).

### 3.4 Paired statistical analysis of PD-L1 expression across specimens

The Kruskal-Wallis test revealed significant differences in both CPS and TPS when comparing preoperative biopsies with surgical resections (p<0.05 for both). Similarly, significant differences were found between surgical resections and metastatic lymph nodes (p<0.05 for both). No statistically significant differences were observed for CPS and TPS between preoperative biopsies and metastatic lymph nodes.

### 3.5 Categorical comparison of PD-L1 expression between primary and metastatic specimens

When comparing paired primary tumors (resection specimens) and metastatic lymph nodes, the overall categorical concordance rate for CPS was 72.1% (49/68). Higher CPS values were observed in the primary tumor in 25.0% (17/68) of cases, while higher CPS was found in the lymph node in 2.9% (2/68) of cases. For TPS, the overall concordance rate was 47.1% (32/68), with higher values in the primary tumor and lymph node in 38.2% (26/68) and 14.7% (10/68) of cases, respectively (**Table 2**).

**Table 2:** PD-L1 expression in primary tumors and metastatic lymph nodes, categorized by CPS and TPS.

Lymph node Primary	CPS <1	CPS 1-19	CPS >20
CPS <1	0 (0%)	0 (0%)	0 (0%)
CPS 1-19	2 (2.9%)	5 (7.4%)	2 (2.9%)
CPS >20	0 (0%)	15 (22.1%)	44 (64.7%)
Lymph node Primary	TPS <1%	TPS 1-49%	TPS >50%
TPS<1%	1 (1.5%)	0 (0%)	0 (0%)
TPS 1-49%	8 (11.8%)	24 (35.3%)	10 (14.7%)
TPS >50%	2 (2.9%)	16 (23.5%)	7 (10.3%)

CPS, Combined positive score, TPS, Tumor proportion score.

### 3.6 Case-wise PD-L1 expression trends

**Figure 5:**
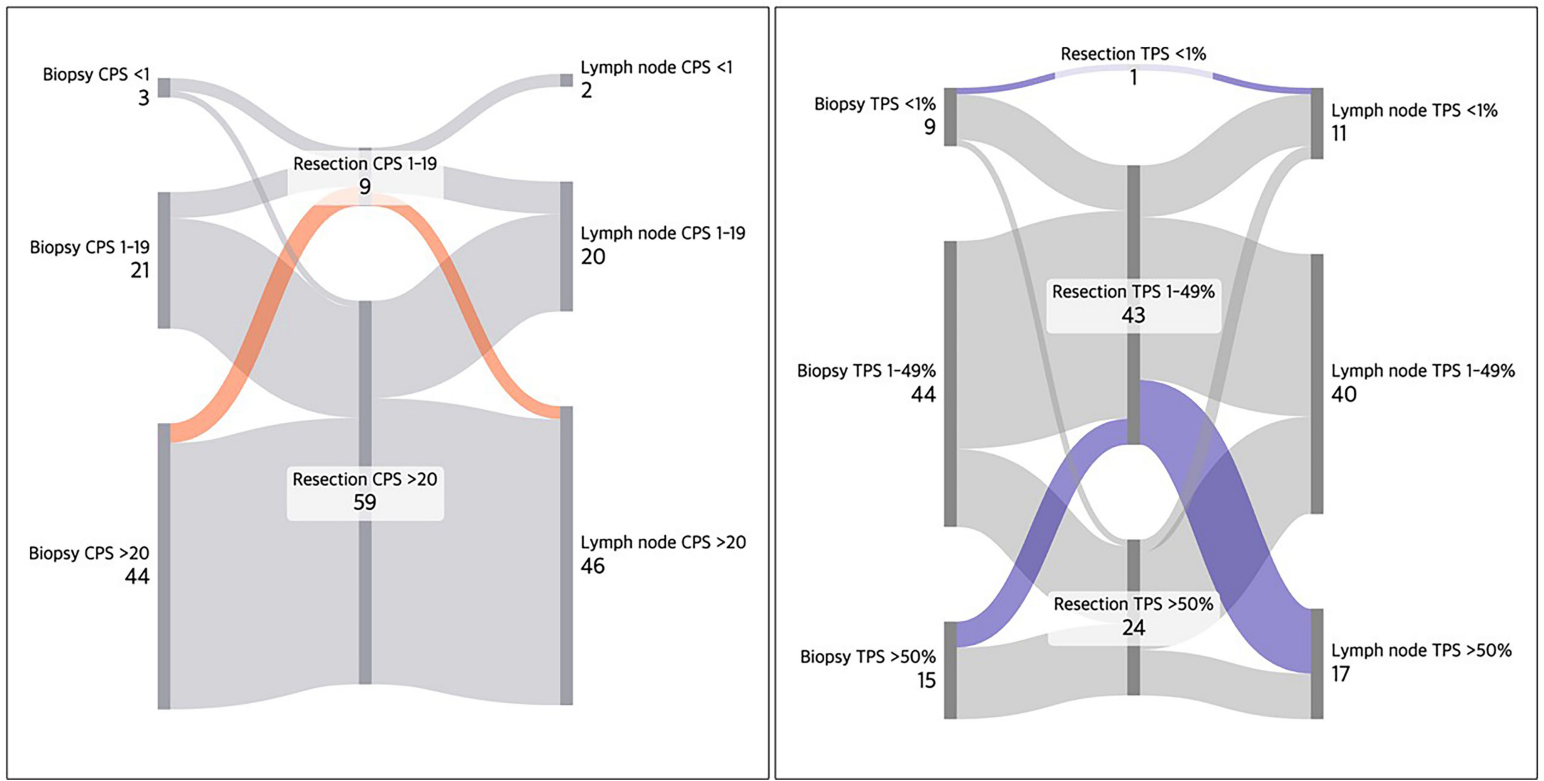
Sankey diagrams showing PD-L1 expression trends (CPS and TPS) across 68 HNSCC cases. Surgical specimens generally exhibited higher CPS (~20 scale) compared to TPS, likely due to PD-L1-positive immune cells. A case demonstrated uniformly low PD-L1 expression (TPS <1%) across all specimens (depicted in top blue). Several cases with inverse PD-L1 expression pattern (lower expression in resection) are also depicted in red orange and blue.

**Figure 5** illustrates case-wise PD-L1 expression trends in CPS and TPS. Average CPS scores in surgical specimens were approximately 20 points higher than corresponding TPS values. One case showed consistently low PD-L1 expression (TPS <1%) across all specimens. Inverse expression patterns, where surgical specimens had lower PD-L1 expression than biopsies or lymph nodes, were observed in a subset of cases (highlighted in orange and blue in **Figure 5**).

## 4 Discussion

This study explores PD-L1 expression patterns in Head and neck squamous cell carcinoma across preoperative biopsies, surgical resections and metastatic lymph nodes, using CPS and TPS categorized into three groups. Oropharyngeal tumors demonstrated clinicopathological features distinct from oral cavity cancers, in part due to their strong association with HPV infection and its correlation with p16 IHC (2). Also, CPS scores from resection specimens were significantly lower in advanced-stage disease compared with early-stage cancer (median value 37.8 vs.67.7, p < 0.05).

### 4.1 CPS versus TPS

Across all specimen types, CPS consistently exceeded TPS, with an average difference of approximately 20 units. This discrepancy reflects the contribution of PD-L1-positive immune cells incorporated into CPS but excluded from TPS. Prior studies have similarly suggested that CPS better represents the tumor microenvironment (11), particularly in surgically resected specimens where immune infiltrates are more comprehensively sampled (12). Notably, inter-specimen concordance was higher for CPS than for TPS (72.1% vs. 48.5%), supporting the notion that CPS provides a more stable and reproducible measure across tumor compartments (13, 14), even though intratumoral heterogeneity remains.

### 4.2 Evaluation of variability of PD-L1 expression

Several studies have highlighted the challenges in evaluating PD-L1 expression in HNSCCs, including variability in expression levels and the difficulty of selecting representative samples (13, 14). Karpathiou et al. reported that PD-L1 expression may decline in archival specimens, potentially compounding heterogeneity in reported results (15). Furthermore, PD-L1 assessment after chemotherapy and/or radiotherapy has yielded conflicting outcomes across studies (16–18).

In other malignancies, such as lung cancer, the consistency of PD-L1 expression between primary tumors and metastases has also been examined. Gradecki et al. observed high concordance between core biopsies and resections in NSCLC, particularly in specimens with diffuse staining involving >50% of tumor cells (19). In contrast, more recent investigations have underscored significant variability in PD-L1 expression across specimen types in both early and advanced NSCLC (6, 20).

### 4.3 Pairwise specimen comparisons of PD-L1 expression

#### 4.3.1 Preoperative biopsy vs. surgical resection

Both CPS and TPS were significantly higher in surgical resections (p < 0.05), consistent with spatial heterogeneity where biopsies may underestimate PD-L1-rich areas. This suggests that interpretations should be done in biopsy and surgical resection parallelly for optimal clinical decision-making, a pattern consistent with precious reports (9).

#### 4.3.2 Surgical resection vs. metastatic lymph node

Significant differences (p < 0.05) were also observed, with lower CPS and TPS in lymph nodes, possibly reflecting temporal heterogeneity arising during the interval of metastasis or recurrence, and/or clonal selection with lower PD-L1 expression. In contrast, Paolino et al. reported higher degree of positivity in metastatic lymph nodes (14). A potential source of discrepancy includes overestimation of PD-L1 expressing cells, which may not truly represent tumor-immune interactions. Taken together, these observations highlight the need for prospective studies comparing primary tumors and matched metastases.

#### 4.3.3 Preoperative biopsy vs. metastatic lymph node

No significant statistical difference was observed overall, though presence of difference at a group level.

#### 4.3.4 Categorical reclassification across specimen types

For CPS, three negative and 21 low-expression preoperative biopsy cases were largely reclassified into the high-expression category in surgical resections. However, in metastatic lymph nodes, 20 cases reverted to low expression, with only 46 maintaining high expression. For TPS, high expression was identified in 15 preoperative biopsies, increasing to 25 surgical resections. In contrast, only 17 metastatic lymph nodes retained this status, while 40 cases shifted to low-expression category. Notably, a category-skipping phenomenon (negative preoperative biopsy to high expressing surgical resection) was observed both in CPS and TPS, including transitions from negative biopsies to high-expression resections, and, conversely, a decline from high-expression resections to negative lymph nodes. These threshold-crossing events underscore the substantial heterogeneity of PD-L1 expression across specimens.

#### 4.3.5 Oropharyngeal cancers vs. oral cavity cancers

When analyzed by anatomical site, high CPS and low TPS groups were the most frequent categories across all specimens from oropharyngeal and oral cavity cancers. Separate Kruskal-Wallis tests revealed that oropharyngeal tumors showed a CPS difference only between preoperative biopsy and surgical resection, while oral cavity tumors demonstrated differences in both CPS and TPS between biopsy and resection, and resection and metastatic lymph node. These findings suggest site-specific biological differences, potentially influenced by HPV status or other tumor–microenvironmental factors, despite their shared squamous histology.

### 4.4 Outliers

Several unique cases were identified. One tongue tumor demonstrated uniformly negative PD-L1 expression across all specimens, suggesting an inherently PD-L1-negative phenotype. Of note, a few cases (oropharynx n=1, oral cavity n=2) displayed inverse patterns, with negative surgical resections but corresponding biopsies or lymph nodes strongly positive. This may reflect heterogeneity of tumors, or clonal selection capable of immune evasion in metastatic sites. These unusual patterns underscore the need for further investigation into the biological behavior of such tumor and their potential clinical implications.

Despite these insights, this study has several limitations. First, it is a retrospective, single-institution study with a relatively small sample size, and the inclusion of a few older archival samples, which may limit generalizability of findings. Second, interobserver variability in PD-L1 scoring, especially for immune cell staining, was not assessed. Third, lymph node specimens collected after resection were not separately analyzed but included as a pooled group, without distinction between normal and metastatic lymph nodes, that could have provided further insight for metastatic potential of tumors. Fourth, we did not correlate PD-L1 expression with clinical outcomes, such as response to immune checkpoint inhibitors, which would be necessary to validate the predictive value of these findings.

## 5 Conclusions

Our study demonstrates that PD-L1 expression in head and neck squamous cell carcinoma is heterogeneous across specimen types. Oropharyngeal tumors generally exhibited overall higher PD-L1 levels, whereas oral cavity tumors showed greater variability in score-based classification across specimens, with statistically significant differences in measured values for both tumor sites. These indicate that no single specimen can reliably represent tumor’s overall PD-L1 status, despite moderate concordance in scoring. Therefore, assessment of multiple specimen types should be considered when determining PD-L1 status for immunotherapy eligibility.

## Data Availability

The raw data supporting the conclusions of this article will be made available by the authors, without undue reservation.
